# Opioid-Free Using Ketamine versus Opioid-Sparing Anesthesia during the Intraoperative Period in Video-Assisted Thoracoscopic Surgery: A Randomized Controlled Trial

**DOI:** 10.3390/jpm14080881

**Published:** 2024-08-21

**Authors:** Hoon Choi, Jaewon Huh, Minju Kim, Seok Whan Moon, Kyung Soo Kim, Wonjung Hwang

**Affiliations:** 1 Department of Anesthesiology and Pain Medicine, Seoul St. Mary’s Hospital, College of Medicine, The Catholic University of Korea, Seoul 06591, Republic of Korea; hoonie83@catholic.ac.kr (H.C.); ether@catholic.ac.kr (J.H.); minju1025@catholic.ac.kr (M.K.); 2 Department of Thoracic and Cardiovascular Surgery, Seoul St. Mary’s Hospital, College of Medicine, The Catholic University of Korea, Seoul 06591, Republic of Korea; swmoon@catholic.ac.kr (S.W.M.); cskks@catholic.ac.kr (K.S.K.)

**Keywords:** opioid-free anesthesia, opioid-sparing anesthesia, thoracic anesthesia, opioid

## Abstract

Opioids effectively manage perioperative pain but have numerous adverse effects. Opioid-free anesthesia (OFA) eliminates intraoperative opioid use; however, evidence for its use in video-assisted thoracoscopic surgery (VATS) is limited. This study assessed the effect of OFA using ketamine in VATS patients compared to opioid-sparing anesthesia (OSA). A total of 91 patients undergoing VATS lobectomy or segmentectomy were randomized to either the OFA group (ketamine) or the OSA group (remifentanil). The primary outcome was the quality of recovery (QoR) on postoperative day (POD) 1, measured with the QoR-40 questionnaire. Secondary outcomes included postoperative pain scores and adverse events. Both groups had comparable baseline and surgical characteristics. On POD 1, the QoR-40 score was higher in the OFA group than in the OSA group (164.3 ± 10.8 vs. 158.7 ± 10.6; mean difference: 5.6, 95% CI: 1.1, 10.0; *p* = 0.015), though this did not meet the pre-specified minimal clinically important difference of 6.3. The visual analog scale score was lower in the OFA group as compared to the OSA group at 0–1 h (4.2 ± 2.3 vs. 6.2 ± 2.1; *p* < 0.001) and 1–4 h after surgery (3.4 ± 1.8 vs. 4.6 ± 1.9; *p* = 0.003). The OFA group had a lower incidence of PONV (2 [4.4%] vs. 9 [19.6%]; *p* = 0.049) and postoperative shivering (4 [8.9%] vs. 13 [28.3%]; *p* = 0.030) than the OSA group at 0–1 h after surgery. Using OFA with ketamine proved feasible, as indicated by the stable intraoperative hemodynamics and absence of intraoperative awareness. Patients undergoing VATS with OFA using ketamine showed a statistically significant, but clinically insignificant, QoR improvement compared to those receiving OSA with remifentanil.

## 1. Introduction

Perioperatively, opioids have long been used to effectively alleviate pain, but their use is now being increasingly scrutinized because of the numerous associated adverse effects [1]. These adverse effects include well-known issues, such as postoperative nausea and vomiting (PONV), shivering, itching, sedation, urinary retention, and gastrointestinal paralysis, in addition to less common but potentially dangerous complications, such as hypotension and chest wall rigidity [2,3]. One of the most significant threats following thoracic surgery is opioid-induced ventilatory impairment, which can result in a prolonged recovery time and delayed tracheal extubation [2]. Opioid-based anesthesia (OBA), especially with remifentanil, can exacerbate postoperative pain and elevate chronic post-surgical pain risk [4]. High intraoperative remifentanil dosages can cause opioid-induced hyperalgesia (OIH), increasing postoperative pain severity and morphine consumption compared to lower or placebo doses [5,6]. Moreover, prolonged perioperative opioid administration, particularly with high doses and post-discharge, raises the risk of persistent postoperative opioid use, contributing to the global opioid epidemic [7]. Furthermore, opioids have been linked to negative oncological outcomes in cancer patients [2].

With the growing awareness of opioid pitfalls, many organizations advocate minimizing perioperative opioid use through balanced anesthesia and multimodal analgesia, known as opioid-sparing anesthesia (OSA) [8,9]. This approach is highlighted by the Early Recovery After Surgery (ERAS) protocol [10]. Opioid-free anesthesia (OFA), which represents the most extreme form of OSA, completely avoids the use of opioids during the intraoperative period [3,11]. The term OFA pertains to the avoidance of opioids throughout the intraoperative period, whereas opioid-free analgesia encompasses an extended period of opioid avoidance during the postoperative phase [2]. Studies have demonstrated the feasibility of the OFA in various types of surgical procedures, including thoracoscopic surgery [11,12,13,14]. Although the application of OFA has the potential to reduce adverse events and enhance patient outcomes, the results have been inconsistent [15]. Overall, the findings show that OFA reduces the incidence of PONV and postoperative complications during the recovery period but does not exhibit clinically significant advantages in terms of postoperative pain or opioid consumption [3,11]. Previous studies on OFA using dexmedetomidine have shown an increased risk of bradycardia [16,17]. Determining the most beneficial surgical procedures and patient populations for the application of OFA, as well as the most effective regimens, is essential [18].

Video-assisted thoracoscopic surgery (VATS) is the preferred treatment for early-stage lung cancer [10,19], and is associated with considerable postoperative pain and a high risk of PONV, leading to a high demand for opioids and poor quality of recovery (QoR) [19,20]. Consequently, patients who undergo a VATS lung resection are suitable candidates for OFA [18]. The QoR-40 questionnaire is a well-established and validated tool used to quantify postoperative recovery and is recommended as an outcome measure by the Standardized Endpoints in Perioperative Medicine (StEP) initiative [21,22]. Although studies on the impact of OFA on patients undergoing VATS with QoR-40 are limited, the results of these studies may provide valuable insights.

Therefore, this study aimed to investigate the effect of OFA using ketamine on QoR in patients who underwent VATS lobectomy or segmentectomy. We compared the most recent and widely used strategies for minimizing intraoperative opioid use: OFA and OSA. We hypothesized that patients with OFA would demonstrate improved QoR-40 scores compared with those receiving OSA. Furthermore, we assessed the feasibility of OFA administration during VATS.

## 2. Materials and Methods

### 2.1. Study Design

This prospective, parallel-group, single-blind (patient) randomized controlled trial was conducted at a tertiary university hospital in accordance with the Declaration of Helsinki. The Institutional Review Board (IRB) of Seoul St. Mary’s Hospital, the Catholic University of Korea, approved the study protocol (KC20MISI0487) on 9 July 2020, and it was registered at the Clinical Research Information Service (CRIS; KCT0005237) prior to patient enrollment on 16 July 2020. The study protocol remained unchanged after the commencement of the trial. All participants provided written informed consent prior to inclusion in the study. This study was reported in accordance with the consolidated standards of reporting trials (CONSORT) 2010 Statement and the CONSORT Patient-Reported Outcomes (CONSORT PRO) Extension [23].

### 2.2. Patients

Eligible participants in our study were patients aged between 20 and 75 years with an American Society of Anesthesiologists (ASA) physical status of I to III who were scheduled for elective VATS lobectomy or segmentectomy under general anesthesia at Seoul St. Mary’s Hospital, the Catholic University of Korea. We excluded patients who had undergone emergency surgery, had chronic pain requiring medication, had a history of neuropsychiatric disease, were unable to communicate, were pregnant or lactating, had contraindications to ketamine or remifentanil, had known allergies, or had a history of adverse events to any of the drugs used for anesthesia.

### 2.3. Randomization and Blinding

Eligible patients were randomly assigned to either the intervention (OFA group) or control (OSA group) group in a 1:1 ratio using a computer-generated allocation sequence with random block sizes of two, four, and six. To conceal the allocation, the group assignments were sealed in opaque, sequentially numbered envelopes created by a researcher who did not participate in the study. The envelope was handed to the participating anesthesiologist one hour before surgery.

Throughout the study, both patients and surgeons were unaware of the group assignments. Similarly, medical staff members responsible for postoperative care and outcome assessment in the post-anesthesia care unit (PACU) and ward were blinded to the group allocations. Although the anesthesiologist performing general anesthesia could not be blinded owing to the different drug administration protocols among the groups, they did not play a role in postoperative care or the assessment of postoperative outcomes.

### 2.4. Intervention

Our institution employs a standardized thoracic anesthesia protocol that conforms to the current ERAS recommendations [10]. Before the commencement of the study, patients received training in assessing their pain levels using a visual analog scale (VAS), with 0 cm denoting no pain and 10 cm indicating the most severe pain conceivable. They were also informed about the use of intravenous (IV) patient-controlled analgesia (PCA). To promote protocol compliance, patients were advised to seek analgesia promptly if their VAS pain scores reached or exceeded 4.

Upon arrival in the operating room, all patients underwent routine monitoring, including electrocardiography, non-invasive blood pressure measurements, pulse oximetry, end-tidal carbon dioxide, neuromuscular monitoring using train-of-four (TOF) stimulation, and bispectral index (BIS; A-2000TM SP, Aspect Medical Systems, Norwood, MA, USA) monitoring of anesthesia depth. Data were recorded every 5 min. Based on their ideal body weight, all patients received an induction dose of IV propofol at 1.5–2.5 mg/kg for general anesthesia. After confirming unconsciousness (BIS value < 60), the absence of an eyelash reflex, and no response to verbal stimulation, a bolus dose of IV rocuronium at 0.6 mg/kg was administered, and after zero TOF twitches were achieved, orotracheal intubation with a left-sided double-lumen endobronchial tube (Human Broncho, Insung Medical Co., Wonju, South Korea) was performed using a direct laryngoscope. Correct positioning of the tube was confirmed by flexible fiberoptic bronchoscopy in both the supine and lateral decubitus positions. All patients received one-lung ventilation according to the protective lung ventilation protocol, which was adjusted at the discretion of the attending anesthesiologist. Anesthesia was maintained using a continuous IV infusion of propofol at 4–12 mg/kg/h to maintain a BIS value between 40 and 60. Muscle relaxation was maintained with a continuous infusion of IV rocuronium at 0.3–0.6 mg/kg/h to maintain a TOF count of 1–2. After the confirmation of an adequate BIS value, patients received a bolus dose of IV nicardipine of 0.5 mg if their systolic blood pressure (SBP) was > 150 mmHg, and a bolus dose of IV ephedrine of 4 mg if their SBP was < 80 mmHg. If the heart rate (HR) increased above 120 bpm, a bolus dose of IV esmolol of 10 mg was administered; if the HR decreased below 50 bpm, a bolus dose of IV atropine of 0.25 mg was administered.

The OFA group was administered a loading dose of IV ketamine at a dose of 0.25 mg/kg immediately after losing consciousness, followed by a continuous infusion at a rate of 0.125–0.25 mg/kg/h [24]. On the other hand, the OSA group received a continuous infusion of IV remifentanil at a dose of 0.5–1.0 μg/kg/min immediately after losing consciousness, which was then adjusted to a rate of 0.05–2.0 μg/kg/min after tracheal intubation. Both drugs were adjusted in steps of 25% to maintain the mean arterial pressure (MAP) within 20% of the baseline value and were discontinued at the end of skin closure. In the OFA group, any intraoperative opioids were prohibited, and in the OSA group, ketamine was not allowed.

To manage postoperative pain, a multimodal analgesic approach involved the administration of IV acetaminophen at 1 g and ketorolac at 30 mg was administered before the incision. These non-opioid analgesics were continued throughout the patient’s hospital stay in the ward. Additionally, an ultrasound-guided thoracic paravertebral block with 20 mL of 0.25% ropivacaine was performed in the fourth intercostal space on the operative side at the end of surgery. To prevent PONV, all patients received IV dexamethasone at 5 mg at the beginning of surgery and IV palonosetron at 75 μg at the end of surgery.

At the end of surgery, all patients were administered IV sugammadex at a dose of 4 mg/kg to reverse neuromuscular blockade. The patients were extubated and moved to the PACU once they demonstrated self-respiration and adequate neuromuscular recovery (TOF ratio ≥ 0.9). If they reported pain with a VAS score ≥ 4 in the PACU, they received an immediate IV dose of fentanyl, in the range of 0.5–1 μg/kg. Once their acute pain was under control, all patients were administered IV PCA, which consisted of fentanyl at 15 μg/kg in 100 mL of normal saline, a basal rate of 0 mL/h, and a bolus of 1 mL, with a lock-out time of 10 min. If a patient experienced PONV, IV metoclopramide at 10 mg was administered. Patients were discharged to the ward when their Aldrete score was ≥ 9. For postoperative pain that could not be controlled with IV PCA throughout the study period, the patients received an IV dose of fentanyl, in the range of 0.5–1 μg/kg.

### 2.5. Primary Outcome

The primary outcome of this study was to evaluate QoR on postoperative day (POD) 1 using the Korean version of the QoR-40 questionnaire [21]. The QoR-40 questionnaire encompasses five dimensions of recovery: physical comfort (12 items), emotional state (9 items), physical independence (5 items), psychological support (7 items), and pain (7 items). Each item is scored on a 5-point Likert scale, ranging from “none of the time” to “all of the time”. The total score on the QoR-40 questionnaire ranges from 40 (the poorest possible recovery) to 200 (the best possible recovery), with a change of 6.3 considered clinically meaningful [25]. The outcome assessor, who was unaware of the group allocations and trained in the use of the QoR-40 questionnaire, interviewed all patients and completed the questionnaire on the day before surgery and on PODs 1 and 2.

### 2.6. Secondary Outcomes

To evaluate the feasibility of OFA in maintaining hemodynamic stability, we collected intraoperative hemodynamic data, including SBP, MAP, HR, and anesthetic depth (BIS), at baseline, before intubation (at the time of unconsciousness), immediately after intubation, at the time of incision, at the end of surgery, and immediately after tracheal extubation. Additionally, episodes of tachycardia (HR > 120 bpm), bradycardia (HR < 45 bpm), hypertension (SBP > 150 mmHg), and hypotension (SBP < 80 mmHg) associated with the administration of esmolol, atropine, nicardipine, and ephedrine were recorded. We also recorded the time to eye opening and extubation from the end of skin closure, and duration of stay in the PACU. Moreover, we evaluated intraoperative awareness in the PACU using a Modified Brice questionnaire [26].

The severity of pain was evaluated using a VAS score, which was measured from the patient’s arrival at the PACU until 48 h after the surgery. The highest VAS score, total PCA consumption, and requirement for rescue analgesics during the time intervals of 0–1, 1–4, 4–24, and 24–48 h were recorded. Additionally, the occurrence of adverse events, such as PONV, shivering, and psychosis, was documented at the same time intervals.

### 2.7. Sample Size

At our institution, the mean QoR-40 score of patients who underwent a VATS lobectomy with OSA on POD 1 was 164 ± 10.1. Given a QoR-40 score of 6.3 as the minimal clinically important difference [25], the necessary sample size to attain a type I error risk of 0.05 and a power of 0.8 in a two-sided test would be 42 patients per group. Accounting for a 10% dropout rate, we determined that 47 patients per group were required and planned to include 94 patients in total. The study was concluded when a predetermined sample size was reached.

### 2.8. Statistical Analysis

A researcher who was blinded to the group allocation conducted a statistical analysis of all randomized patients using an intention-to-treat approach. The normality of the distribution of quantitative variables was assessed using the Kolmogorov–Smirnov test. An independent-sample t-test or Mann–Whitney U test was used to analyze quantitative variables, including the primary outcome (QoR-40 score on POD 1). Chi-squared or Fisher’s exact tests were used to analyze qualitative variables. For sensitivity analysis, the analysis of covariance (ANCOVA) was performed after controlling for preoperative QoR-40 scores. Values are expressed as the mean ± standard deviation or number of patients (percentage). All statistical analyses were performed using two-tailed tests, and a *p* value of less than 0.05 was considered statistically significant. Statistical analyses were performed using SPSS software (version 24.0; IBM SPSS Inc., Armonk, NY, USA).

## 3. Results

### 3.1. Patients

**Figure 1:**
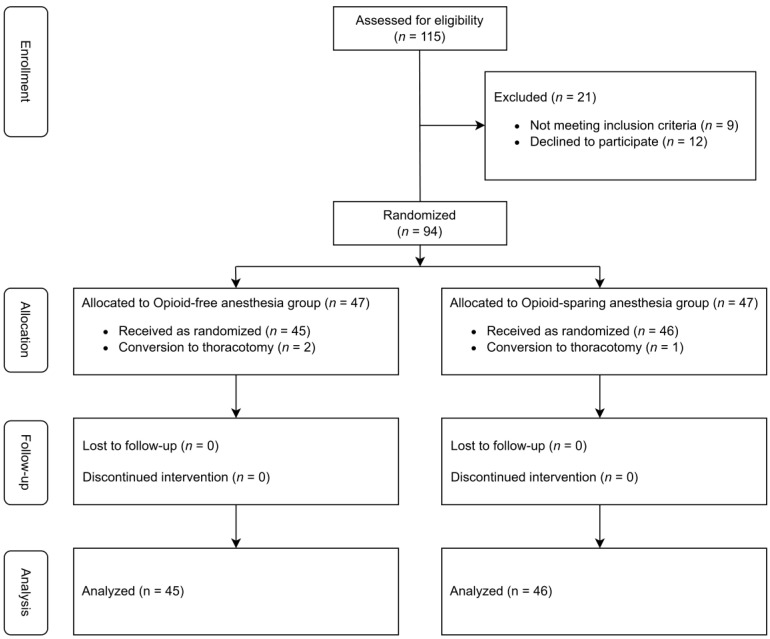
Consolidated standards of reporting trials (CONSORT) flowchart of the study.

Between September 2020 and May 2022, 115 patients were screened for the study and 94 were randomly assigned to receive either OFA or OSA. However, three patients (two in the OFA group and one in the OSA group) were excluded from the analysis because of a conversion to thoracotomy during surgery. Therefore, 91 patients (45 and 46 in the OFA and OSA groups, respectively) were included in the final analysis (Figure 1).

The baseline characteristics and surgical details of the patients are presented in Table 1. The patient demographics, underlying conditions, and surgical procedures were consistent across all study participants.

### 3.2. Primary Outcome

All the patients successfully completed the QoR-40 questionnaire without encountering any difficulties (Table 2). The baseline QoR-40 score was comparable between the two groups, with no statistically significant difference observed. The primary outcome of the study, the QoR-40 score on POD 1, was found to be significantly higher in the OFA group than in the OSA group (164.3 ± 10.8 vs. 158.7 ± 10.6; mean difference: 5.6, 95% CI: 1.1, 10.0; *p* = 0.015). However, the mean difference did not meet the pre-specified minimal clinically important difference of 6.3 [25]. When comparing the five domains of the QoR-40 questionnaire on POD 1, the OFA group scored higher in the physical comfort domain and pain domain than the OSA group. Similar results were observed on POD 2. The QoR-40 score on POD 2 was significantly higher in the OFA group than in the OSA group, but the mean difference was less than 6.3. The score in the physical comfort domain was higher in the OFA group, while the score in the pain domain was not different.

### 3.3. Secondary Outcomes

**Figure 2:**
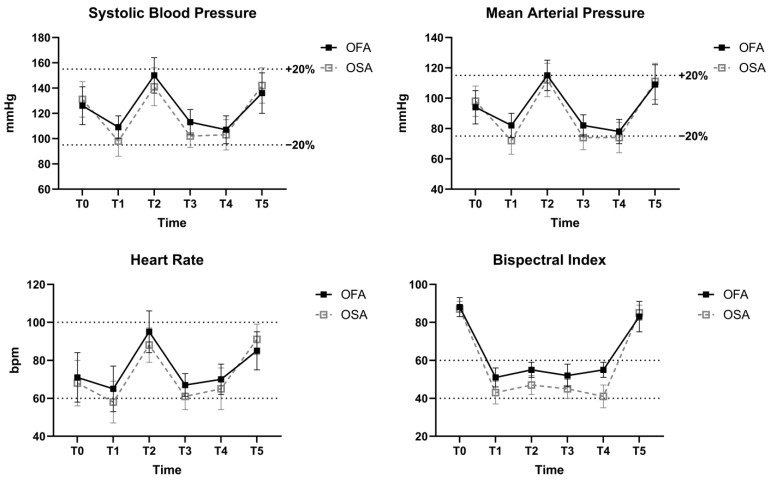
Intraoperative hemodynamic and anesthetic depth data. T0, baseline; T1, before intubation (at the time of unconsciousness); T2, immediately after intubation; T3, at the time of incision; T4, at the end of surgery; T5, immediately after tracheal extubation.

Table 3 compares the secondary outcomes between the two groups. The time to eye opening was significantly longer in the OFA group than in the OSA group. Furthermore, the time to extubation was significantly longer in the OFA group than in the OSA group. Additionally, the duration of stay in the PACU was also significantly longer in the OFA group than in the OSA group. With regard to intraoperative complications, the incidence of hemodynamic disturbance was similar between the two groups, and there were no instances of intraoperative awareness. During surgery, the SBP was maintained within 20% of the baseline value, and the BIS value was consistently between 40 and 60 (Figure 2).

The VAS score was significantly lower in the OFA group as compared to the OSA group at 0–1 h and 1–4 h after surgery. Moreover, the OFA group used less PCA at 0–1 h and 1–4 h after surgery as compared to the OSA. The number of patients requiring rescue analgesics was significantly lower in the OFA group than in the OSA group at 0–1 h. However, the effects of OFA on postoperative pain were not sustained at later time intervals (4–24 and 24–48 h).

The incidence of adverse events differed only 0–1 h after surgery. There was a significant difference in the incidence of PONV and postoperative shivering between the two groups at 0–1 h after surgery, with the incidence being lower in the OFA group. However, at later time intervals, such as 1–4 h, 4–24 h, and 24–48 h, the occurrence of PONV and postoperative shivering were similar between the two groups. None of the patients experienced psychosis during the study period.

### 3.4. Sensitivity Analysis

ANCOVA was conducted to evaluate the effect of OFA on the QoR-40 score on POD 1 after accounting for the preoperative QoR-40 score. The results show a statistically significant difference in the QoR-40 scores between the two groups after adjusting for the preoperative QoR-40 score (*p* = 0.035). However, the adjusted mean difference was 4.4, which was less than the pre-specified minimal clinically important difference of 6.3 [25].

## 4. Discussion

The main aim of this study was to evaluate the effect of OFA with ketamine on the QoR in patients who underwent VATS lobectomy or segmentectomy. Our results suggest that OFA did not result in a clinically significant improvement in QoR-40 scores compared to OSA. Although OFA showed enhancements in the patient comfort and pain domains, these benefits were not reflected in the overall QoR-40 scores on PODs 1 and 2. Furthermore, patients who received OFA required a longer time to wake up from anesthesia than those who received OSA. Nonetheless, the use of OFA with ketamine in a VATS lobectomy or segmentectomy is feasible, as evidenced by stable intraoperative hemodynamics and the absence of intraoperative awareness. Additionally, patients who underwent OFA experienced less pain up to 4 h after surgery and had fewer occurrences of PONV and postoperative shivering in the PACU than those who underwent OSA.

While it is important to consider separate outcomes, such as the pain score and incidence of PONV, the overall QoR, as perceived by the patient, appears to be of even greater significance. Various QoR scores have been developed to capture these outcomes; however, only a few have been extensively validated across a wide range of surgical settings. QoR-40 has been extensively validated and recommended by the Standardized Endpoints in Perioperative Medicine (StEP) initiative [21,22]. This score has been associated with postoperative complications in the first month after surgery, which is also a relevant outcome for the evaluation of anesthesia protocols [27]. In a previous study that compared OFA with OBA in patients undergoing major surgery (not including thoracic surgery), OFA resulted in a statistically significant but clinically insignificant improvement in QoR [28]. Although the drug regimens used in this study were different (clonidine, magnesium, and lidocaine in the OFA group, and ketamine in both groups), the minimal effect of OFA on QoR was similar. Additionally, a study in patients undergoing VATS that compared OFA using dexmedetomidine with OBA reported a statistically significant improvement; however, it also fell short of the pre-specified minimal clinically important difference [14]. In another multicenter study involving patients who had undergone endoscopic sinus surgery, comparable results were reported for QoR-40 [29]. Additional research is necessary to assess the potential influence of OFA on the overall QoR.

The failure of the OFA group to produce clinically significant improvements in QoR may be attributed to the fact that the control group received OSA. This study differs from previous research that focused on comparing OFA to OBA [12,13,14,16,30,31,32] by utilizing a control group (the OSA group) that strictly adhered to our institutional thoracic anesthesia protocol, which included patient education, anti-inflammatory medications, and regional analgesia, in accordance with the current ERAS recommendations [10]. Remifentanil, which was administered to patients in the OSA group, is a highly potent opioid; however, the average dose of remifentanil used in our study was 0.12 ± 0.05 μg/kg/min, which is lower than the previous studies conducted in VATS that reported an average dose of 0.14 ± 0.9 μg/kg/min [33]. Although previous research has compared OFA to OSA in patients undergoing total hip arthroplasty, our study is the first to assess the overall QoR between these two groups in patients undergoing VATS [34].

We performed OFA with ketamine in patients who underwent VATS to identify the ideal regimen and the most appropriate surgery. The approach taken by OFA typically entails the use of a multimodal regimen [3,11], which adds complexity, increases resource and cost requirements, and causes potential adverse effects without significant advantages [2]. For instance, dexmedetomidine can result in airway collapse [35] and has shown controversial results in terms of bradycardia and hypotension [2,36], whereas anti-inflammatory medications can cause gastrointestinal and acute renal failure [37]. Although numerous previous studies used multiple agents, including dexmedetomidine [12,13,14,16,17,28,30,31,32], this study eliminated the need for extensive resources by substituting remifentanil with ketamine alone. While one study stopped prematurely because of the occurrence of severe bradycardia after administering a high dose of dexmedetomidine [17], we utilized a sub-anesthetic dose of ketamine and discovered that OFA with ketamine is both feasible and safe, as evidenced by stable intraoperative hemodynamics and the absence of intraoperative awareness and postoperative psychosis. The incidence of PONV in patients undergoing VATS can be as high as 50% [38], and opioid-induced ventilatory impairment can be life-threatening owing to reduced lung capacity and the resulting pulmonary inflammation and edema after surgery [19]. Although VATS is associated with less postoperative pain than thoracotomy, it is still considered severe [19,20]. However, the use of OFA has been limited in thoracic surgery, as evidenced by the absence of thoracic surgery in the meta-analyses conducted [16,30,31,32]. Few studies have examined OFA using dexmedetomidine in comparison with OBA in patients undergoing VATS [12,13,14], and to the best of our knowledge, this study is the first to compare OFA using only ketamine with OSA in patients undergoing VATS.

Although the effect of OFA on overall recovery, as evaluated by the QoR-40 questionnaire, was not found to be clinically significant, certain outcomes, such as postoperative pain, as measured by the VAS score and PCA consumption, and the incidence of adverse effects, including PONV and postoperative shivering, demonstrated favorable results. These findings are consistent with previous meta-analyses [16,30,31,32]. Studies have indicated that short-acting opioids, such as remifentanil, may result in the rapid development of opioid tolerance and OIH [39]. A meta-analysis revealed that the intraoperative remifentanil dose was associated with postoperative pain scores, hyperalgesia, time to first postoperative analgesic requirement, and overall postoperative analgesic requirements [6]. The use of opioids after surgery is recognized as one of four risk factors for PONV according to the Apfel simplified risk score. Furthermore, both intraoperative and postoperative administrations of opioids have been associated with an increased risk of PONV, with the likelihood increasing in proportion to the dosage [40]. Postoperative shivering is often considered a result of adrenergic activation due to acute opioid withdrawal. This phenomenon is associated with opioid-induced hyperalgesia (OIH) and acute tolerance, particularly when short-acting opioids, such as remifentanil, are used [41]. In our study, during the early postoperative period, postoperative pain, the incidence of PONV, and postoperative shivering were consistently higher in the OSA group than in the OFA group. The observed decreases in pain, postoperative PONV, and shivering incidents following the OFA protocol could be attributed to the direct elimination of intraoperative opioids, reduction in postoperative opioid use, and the impact of ketamine [42,43]. Our results are consistent with those of a previous study reporting a reduced incidence of PONV in patients who underwent VATS. Although postoperative pain scores and opioid consumption were comparable between OFA and OBA in the same study, the majority of patients underwent wedge resection, where pain levels may have been too low to show any difference compared with our study population [12]. Additionally, one study of patients undergoing VATS reported no differences in the incidence of PONV and postoperative pain scores; however, dexmedetomidine was used in both groups [13].

The impact of ketamine on postoperative pain and neurocognitive disorders should be emphasized. Ketamine is particularly useful in patients who may have high opioid requirements as it reduces postoperative opioid use, hyperalgesia, and post-surgical pain, especially in opioid-tolerant patients [43,44]. In a recent study on the effects of ketamine on pain outcomes after thoracotomy, the group that received ketamine showed significantly lower VAS scores than the control group, consistent with the findings of this study [45]. However, another study on the effects of ketamine on outcomes in critically ill patients did not demonstrate the superiority of the ketamine group over the control group in terms of mortality, pain, opioid consumption, midazolam consumption, or duration of stay in the ICU. Nevertheless, the ketamine group in this study exhibited less delirium than the control group [46]. Although ketamine-induced psychosis was evaluated as a secondary outcome in our study, further research is needed to investigate the impact of ketamine in different situations and its effect on postoperative neurocognitive disorders.

Our study had several limitations. First, the depth of anesthesia monitoring during OFA has not been validated. According to one study, when BIS monitoring is employed with ketamine, the threshold of anesthesia should be raised by 10% of the highest value for the range [47]. In our study, BIS values of 40–60 were used to indicate sufficient anesthesia, which might have led to excessive anesthesia in the OFA group. Second, we only employed an opioid-free technique during the intraoperative period. The most common side and long-term adverse effects of opioids are associated with postoperative use [11]. The benefits of opioid avoidance may be more significant when opioids are administered throughout the perioperative period [2]. However, opioid-free analgesia in thoracic surgery has not yet been validated, and the complete elimination of postoperative opioids in surgeries associated with severe postoperative pain may be unethical. Third, remifentanil, an ultra-short-acting opioid used intraoperatively in the OSA group, may have had minimal postoperative effects. The effects of intraoperative opioids may have been more profound if longer-acting opioids were used. However, remifentanil was selected because it is the most widely used intraoperative opioid in South Korea and the most suitable for early recovery [10]. In addition, the intraoperative dose of remifentanil was associated with postoperative pain scores, hyperalgesia, time to first postoperative analgesic requirement, and postoperative analgesic requirements [6]. Fourth, ethical considerations led to the exclusion of pediatric and geriatric populations as well as patients with severe systemic diseases (ASA physical status IV). Further research on these groups is required.

## 5. Conclusions

In conclusion, patients who underwent a VATS lobectomy or segmentectomy and received OFA with ketamine showed a statistically significant, but not clinically significant, improvement in QoR compared with those who received OSA with remifentanil. Nonetheless, OFA was superior in terms of pain management, incidence of PONV, and postoperative shivering. Moreover, the OFA with ketamine is a viable and safe option for patients undergoing a VATS lobectomy or segmentectomy. To determine the most effective OFA protocol in terms of both efficacy and cost, future clinical trials should focus on identifying the patient population and surgical procedures for which OFA would provide the greatest benefit. OFA may also open new avenues for personalized medicine as certain patients and/or surgeries are at a higher risk of opioid-related adverse effects.

## Figures and Tables

**Table 1:** Baseline and surgical characteristics. Values are expressed as mean ± standard deviation, or number of patients (percentage).

	OFA Group (*n* = 45)	OSA Group (*n* = 46)
Age (year)	57 ± 7	58 ± 6
Gender (male/female)	22/23	24/22
BMI (kg/m^2^)	21.8 ± 2.9	22.5 ± 2.6
Current smoker	12 (26.7%)	8 (17.4%)
ASA physical status (I and II/III)	38/7	41/5
Hypertension	7 (15.6%)	11 (23.9%)
Diabetes mellitus	5 (11.1%)	7 (15.2%)
Operation side (right/left)	25/20	29/17
Blood loss (mL)	95 ± 41	102 ± 43
Working window size (cm)	4.6 ± 0.5	4.5 ± 0.4
Duration of surgery (min)	172 ± 33	164 ± 31

OFA, opioid-free anesthesia; OSA, opioid-sparing anesthesia; BMI, body mass index; ASA, American Society of Anesthesiologists.

**Table 2:** Primary outcome analysis. Values are presented as mean ± standard deviation.

	OFA Group (*n* = 45)	OSA Group (*n* = 46)	Mean Difference (95% CI)	*p* Value
Preoperative QoR-40				
Total	183.5 ± 12.7	180.2 ± 13.7	3.4 (−2.2, 8.9)	0.229
Physical comfort	55.8 ± 3.3	55.0 ± 3.4	0.8 (−0.6, 2.2)	0.251
Emotional state	40.7 ± 3.9	39.9 ± 4.0	0.8 (−0.8, 2.5)	0.326
Physical independence	21.4 ± 3.3	20.7 ± 3.0	0.7 (−0.6, 2.0)	0.286
Psychological support	33.0 ± 2.8	32.1 ± 3.1	0.9 (−0.3, 2.2)	0.138
Pain	32.6 ± 2.6	32.5 ± 2.6	0.1 (−1.0, 1.2)	0.854
QoR-40 on postoperative day 1				
Total	164.3 ± 10.8	158.7 ± 10.6	5.6 (1.1, 10.0)	0.015
Physical comfort	50.5 ± 4.6	47.8 ± 5.1	2.7 (0.7, 4.8)	0.009
Emotional state	35.2 ± 3.4	35.4 ± 2.9	−0.2 (−1.5, 1.1)	0.772
Physical independence	17.7 ± 3.3	17.6 ± 3.2	0.1 (−1.2, 1.5)	0.830
Psychological support	31.4 ± 3.2	30.7 ± 2.8	0.6 (−0.6, 1.9)	0.332
Pain	29.5 ± 3.4	27.2 ± 3.8	2.2 (0.7, 3.8)	0.004
QoR-40 on postoperative day 2				
Total	168.1 ± 14.2	162.2 ± 13.6	6.0 (0.2, 11.8)	0.044
Physical comfort	50.9 ± 5.0	48.3 ± 4.9	2.6 (0.6, 4.7)	0.012
Emotional state	36.0 ± 3.8	35.7 ± 3.3	0.3 (−1.2, 1.8)	0.685
Physical independence	19.4 ± 3.7	18.3 ± 3.3	1.1 (−0.4, 2.6)	0.142
Psychological support	30.3 ± 4.9	28.9 ± 4.8	1.4 (−0.6, 3.4)	0.173
Pain	31.6 ± 3.8	31.0 ± 3.6	0.5 (−1.0, 2.1)	0.490

OFA, opioid-free anesthesia; OSA, opioid-sparing anesthesia; CI, confidence interval; QoR, quality of recovery.

**Table 3:** Secondary outcome analysis. Values are presented as mean ± standard deviation, or number of patients (percentage).

	OFA Group (*n* = 45)	OSA Group (*n* = 46)	Mean Difference or RR (95% CI)	*p* Value
Intraoperative				
Duration of anesthesia (min)	221 ± 38	209 ± 32	12 (−3, 27)	0.107
Time to eye opening (s)	438 ± 102	306 ± 96	132 (91, 173)	<0.001
Time to extubation (s)	492 ± 126	342 ± 104	150 (102, 198)	<0.001
Propofol (mg/kg/h)	8.4 ± 1.6	8.7 ± 1.9	−0.3 (−1.0, 0.4)	0.418
Ketamine (mg/kg/h)	0.27 ± 0.02	-	-	-
Remifentanil (μg/kg/min)	-	0.12 ± 0.05	-	-
Hemodynamic disturbance	13 (28.9%)	22 (47.8%)	0.6 (0.3, 1.0)	0.063
Intraoperative awareness	0 (0.0%)	0 (0.0%)	-	>0.999
0–1 h after surgery				
PACU duration (min)	47 ± 6	39 ± 8	8 (5, 11)	<0.001
VAS	4.2 ± 2.3	6.2 ± 2.1	−2.0 (−2.9, −1.1)	<0.001
PCA consumption (mL)	1.5 ± 1.1	2.3 ± 1.2	−0.8 (−1.3, −0.3)	0.001
Any postoperative opioid ^1^	22 (48.9%)	31 (67.4%)	0.7 (0.5, 1.0)	0.073
Rescue opioid ^2^	16 (35.6%)	30 (65.2%)	0.5 (0.3, 0.8)	0.004
Nausea and vomiting	2 (4.4%)	9 (19.6%)	0.2 (0.1, 0.9)	0.049
Shivering	4 (8.9%)	13 (28.3%)	0.3 (0.1, 0.8)	0.030
Psychosis	0 (0.0%)	0 (0.0%)	-	>0.999
1–4 h after surgery				
VAS	3.4 ± 1.8	4.6 ± 1.9	−1.2 (−2.0, −0.4)	0.003
PCA consumption (mL)	4.7 ± 2.6	6.5 ± 2.7	−1.8 (−2.9, −0.7)	0.002
Any postoperative opioid ^1^	25 (55.6%)	32 (69.6%)	0.8 (0.6, 1.1)	0.167
Rescue opioid ^2^	2 (4.4%)	5 (10.9%)	0.4 (0.1, 1.7)	0.435
Nausea and vomiting	2 (4.4%)	4 (8.7%)	0.5 (0.1, 2.3)	0.677
Shivering	0 (0.0%)	2 (4.3%)	0.0 (0.0, 1.9)	0.495
Psychosis	0 (0.0%)	0 (0.0%)	-	>0.999
4–24 h after surgery				
VAS	4.5 ± 2.0	5.0 ± 2.3	−0.5 (−1.4, 0.4)	0.272
PCA consumption (mL)	14.8 ± 12.3	17.7 ± 11.4	−2.9 (−7.8, 2.0)	0.246
Any postoperative opioid ^1^	45 (100%)	46 (100%)	1.0 (0.9, 1.1)	>0.999
Rescue opioid ^2^	9 (20.0%)	13 (28.3%)	0.7 (0.3, 1.5)	0.358
Nausea and vomiting	4 (8.9%)	8 (17.4%)	0.5 (0.2, 1.5)	0.354
Shivering	0 (0.0%)	1 (2.2%)	0.0 (0.0, 3.9)	>0.999
Psychosis	0 (0.0%)	0 (0.0%)	-	>0.999
24–48 h after surgery				
VAS	4.3 ± 2.6	4.4 ± 2.7	−0.1 (−1.2, 1.0)	0.858
PCA consumption (mL)	16.3 ± 10.7	20.1 ± 14.3	−3.8 (−9.1, 1.5)	0.155
Any postoperative opioid ^1^	44 (97.8%)	43 (93.5%)	1.0 (0.9, 1.2)	0.617
Rescue opioid ^2^	11 (24.4%)	12 (26.1%)	0.9 (0.5, 1.9)	0.857
Nausea and vomiting	3 (6.7%)	7 (15.2%)	0.4 (0.1, 1.5)	0.316
Shivering	0 (0.0%)	0 (0.0%)	-	>0.999
Psychosis	0 (0.0%)	0 (0.0%)	-	>0.999

^1^ Patients requiring postoperative opioid, including the PCA. ^2^ Patients requiring postoperative rescue opioid other than the PCA. OFA, opioid-free anesthesia; OSA, opioid-sparing anesthesia; RR, relative risk; CI, confidence interval; PACU, post-anesthesia care unit; VAS, visual analog scale; PCA, patient-controlled analgesia.

## Data Availability

The data presented in this study are available on request from the corresponding authors.
